# Diarrhoeal diseases among adult population in an agricultural community Hanam province, Vietnam, with high wastewater and excreta re-use

**DOI:** 10.1186/1471-2458-14-978

**Published:** 2014-09-20

**Authors:** Phuc Pham-Duc, Hung Nguyen-Viet, Jan Hattendorf, Phung Dac Cam, Christian Zurbrügg, Jakob Zinsstag, Peter Odermatt

**Affiliations:** Center for Public Health and Ecosystem Research (CENPHER), Hanoi School of Public Health (HSPH), 138 Giang Vo, Hanoi, Vietnam; Department of Epidemiology and Public Health, Swiss Tropical and Public Health Institute, P.O. Box, CH-4002, Basel, Switzerland; University of Basel, P.O. Box, CH-4003, Basel, Switzerland; National Institute of Hygiene and Epidemiology, 1 Yersin, Hanoi, Vietnam; Sandec - Department of Water and Sanitation in Developing Countries, Swiss Federal Institute of Aquatic Science and Technology, P. O. Box, CH-8600, Dübendorf, Switzerland

**Keywords:** Diarrhoeal disease, Wastewater, Excreta, Agriculture, Vietnam

## Abstract

**Background:**

Despite the potential health risks of wastewater and excreta use as fertiliser in agriculture, it is still widespread in Vietnam. However, the importance of diarrheal risk in adults’ associated with the combined exposures to both excreta and wastewater use in agriculture is largely unknown. This study was carried out to determine diarrhoeal incidence and associated risk factors among the adult population exposed to wastewater and excreta used in agriculture in Hanam province, Vietnam.

**Methods:**

An open cohort of 867 adults, aged 16–65 years, was followed weekly for 12 months to determine the incidence of diarrhoea. A nested case–control study was used to assess the risk factors of diarrhoeal episodes. Two hundred and thirty-two pairs of cases and controls were identified and exposure information related to wastewater, human and animal excreta, personal hygiene practices, and food and water consumption was collected.

**Results:**

The incidence rate of reported diarrhoea was 0.28 episodes per person-years at risk. The risk factors for diarrhoeal diseases included direct contact with the Nhue River water (odds ratio [OR] = 2.4, attributable fraction [AF] 27%), local pond water (OR = 2.3, AF 14%), composting of human excreta for a duration less than 3 months (OR = 2.4, AF 51%), handling human excreta in field work (OR = 5.4, AF 7%), handling animal excreta in field work (OR = 3.3, AF 36%), lack of protective measures while working (OR = 6.9, AF 78%), never or rarely washing hands with soap (OR = 3.3, AF 51%), use of rainwater for drinking (OR = 5.4, AF 77%) and eating raw vegetables the day before (OR = 2.4, AF 12%).

**Conclusions:**

Our study shows that professional exposure to wastewater and excreta during agricultural activities are significantly contributing to the risk of diarrhoea in adults. The highest attributable fractions were obtained for direct contact with Nhue River and local ponds, handling practices of human and animal excreta as fertilisers, lack of protective measures while working and poor personal hygiene practices, and unsafe food and water consumption were associated with the risk of diarrhoeal episodes in adults. Improve personal hygiene practices and use of relevant treated wastewater and excreta as the public health measures to reduce these exposures will be most effective and are urgently warranted.

## Background

In resource-poor countries, wastewater is used as a source of crop nutrients and reliable irrigation [1–5]. Wastewater use in agriculture has substantial benefits, but can also pose substantial risks to public health, in particular when untreated wastewater is used for crop irrigation. Farmers often have no alternative but to use untreated wastewater because there is no wastewater treatment and freshwater is either unavailable or too expensive. The major risks to public health are microbial and chemical. Wastewater use in agriculture can also create environmental risks in the form of soil and groundwater pollution [6]. In some Asian countries, including Vietnam, the use of excreta for increasing crop yields and fish production is indeed very common [7]. Despite the potential health risks of excreta use as fertiliser in agriculture, it is still widespread in northern and central Vietnam [8, 9]. Health hazards associated with the use of wastewater and excreta in agriculture and aquaculture are significant health concerns in developing countries [7, 10, 11].

Amongst these health hazards, diarrhoeal disease remains one of the most important environmental health problems in resource-poor countries [10, 11]. Diarrhoeal disease is the third leading cause of death in low-income countries, killing an estimated 1.8 million people every year, most of which occur in children under the age of five [12]. Murray et al. [13] indicated that diarrhoea was a major cause of the burden accounting for 3.6% of global Disability-Adjusted Life Years (DALYs). The occurrence of gastrointestinal diseases, including diarrhoea, has been associated with the consumption of wastewater-irrigated vegetables [14]. High-risk groups of people for these diseases are farmers with prolonged wastewater contact, their families, and nearby communities exposed to wastewater irrigation [10]. For example, diarrhoeal disease was observed with significantly higher prevalence in people exposed to wastewater in Pakistan [15]. In addition, a study in Mexico found a higher prevalence of diarrhoeal disease in children under 5 years of age exposed to untreated wastewater than those who were exposed to wastewater retained in a single reservoir or no irrigated wastewater at all [16]. In Vietnam, an epidemiological study showed that close contact with wastewater was also associated with a risk of diarrhoeal disease in adults [17]. However, the importance of diarrheal risk in adults’ related handling practices of human and animal excreta is largely unknown as very few studies have assessed the risk of diarrhoeal diseases associated with the combined exposures to both excreta and wastewater use in agriculture and aquaculture in this population group.

The purpose of our study was to identify risk factors for diarrhoeal episodes in adult farmers in Northern Vietnam. First, we assessed the incidence of diarrhoeal episodes among adults living and working in an agricultural community, where human and animal excreta and wastewater is intensively used to irrigate field and fish feeding. Then, risk factors, including sanitary conditions, drinking water, food consumption, and personal hygiene practices were identified with a nested case–control study.

## Methods

### Study area

**Figure 1:**
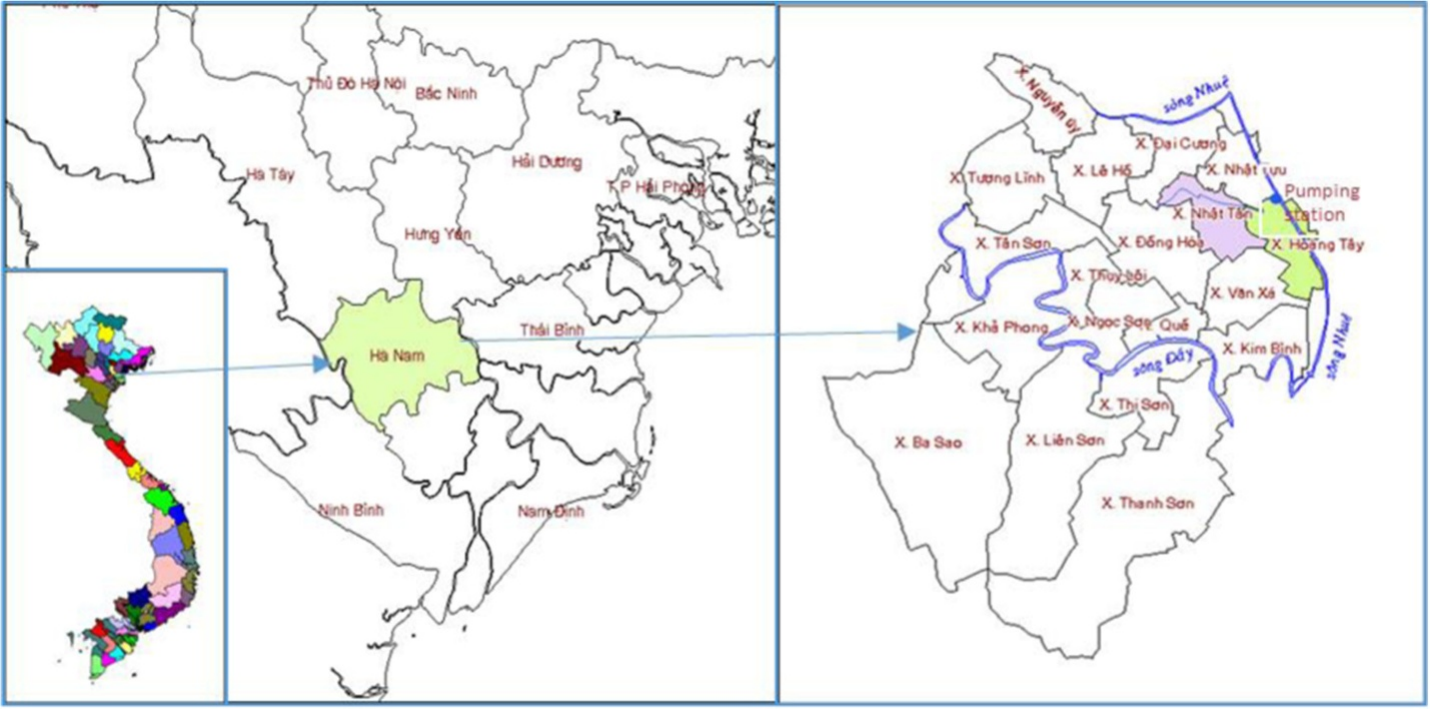
**Map of the study sites in Hoang Tay and Nhat Tan communes, Hanam province, northern Vietnam.**

The study was carried out in Nhat Tan and Hoang Tay communes in King Bang district, Hanam province (20.32° N, 105.54° E), Northern Vietnam, situated about 60 km south of Hanoi (Figure 1). Hanam is located at the Red River Delta with a climate characterized by a tropical monsoon climate, hot and humid. The annual average temperature is around 23-24°C, the average number of hours of sunshine around 1,300-1,500 hours/year. The annual average rainfall is 1,900 mm, in the rainy season is 233.3 mm (April - October) and in the dry season is 39.8 mm (January - March and November - December). The annual average humidity is 85%, the monthly average humidity is highest in March (95.5%) and lowest in November (82.5%) [18, 19]. The number of inhabitants was about 10,500 (2,700 households) and 5,700 (1,600 households) in Nhat Tan and Hoang Tay communes, respectively. Most households raise livestock in their compounds (e.g., chickens, ducks, and pigs). The residential areas are in the vicinity of fields used for rice cultivation, vegetable planting, and fish breeding. Rice fields and local ponds cover about 50% of the residential areas. Hoang Tay commune border the Nhue River and the Nhat Tan commune is connected with Nhue River through the pump stations and canal systems. Nhue River was received Hanoi’s wastewater originating from households, industry, and other sources such as hospitals, is directly discharged without any treatment [20]. The river water is used for crop irrigation and in fish ponds. Several pumping stations are located along the river and a system of open and closed canals distribute the water to the local fields and fish ponds. Wastewater from households (grey water from kitchens and bathrooms and effluent from septic tanks and sanitation facilities) is directly discharged into the small irrigation canals. The area has two main rice production cycles per year, one called “spring season” from January to June and the other “autumn season” from July to October. Human and animal excreta are used as fertilizer in Hanam, as in many other places in northern and central Vietnam. In general, excreta from double or single vault latrines are not or only partially composted before re-use on the fields. Personal protective measures to prevent contamination are often lacking.

### Study design

The recruited study subjects were adults of both sexes, aged 16–65 years from the 405 randomly selected households, who participated in the baseline surveys in 2008 and 2009 [21]. The characteristics of the surveyed households were described in Table 1. A total of 867 subjects were followed from August 2009 to July 2010, the duration that each person was under observation. The participants were followed from enrolment to the end of the study, or until their withdrawal due to various reasons (i.e., deaths, morbidity, or not willing to continue).

**Table 1:** **Characteristics of the study households (N = 405) in Nhat Tan and Hoang Tay communes, Hanam province, Vietnam, 2009-2010**

No	Characteristics of the surveyed household	N (%)
1	Socio-economic status	
	- poor households	134 (33)
2	Water sources and sanitary and hygiene conditions	
	- tap water	180 (44)
	- drilled tube well water	255 (63)
	- rainwater use	351 (87)
	- single vault latrine	225 (56)
	- septic tank	129 (32)
	- poor sanitary condition	143 (35)
3	Exposed to human and animal excreta	
	- composting of human excreta > 3 months before use	131 (32)
	- use of human excreta as fertiliser in agriculture	208 (51)
	- animals breeded	341 (84)
	- animal excreta use as fertiliser in agriculture	175 (43)
4	Exposed to Nhue River water	
	- Nhue River water use for field irrigation	375 (93)

Each participant was assigned an identification number. All related household characteristics (i.e., socio-economic status [SES] and sanitary condition; type of water source; latrine type in the household; and animal husbandry) and personal characteristics (i.e., age, sex, education, occupation) were collected in the baseline surveys. The participants were visited weekly by trained village health workers who collected the past weeks’ information on diarrhoeal episodes. Variables describing personal characteristics as well as the household’s SES, sanitary conditions, water source and type of latrine use in the household, and animal husbandry obtained from the baseline surveys were also included in the analyses [22]. The assumption was held that these variables remained unchanged during the study period.

The household’s economic status were assessed with a list of indicators which included surface of household’s rice field and fish ponds, number of animals (pig, chickens, ducks, buffalos, cows, dogs and cats), housing characteristics (building materials, number of bedrooms), and household assets (motorbike, bicycle, refrigerator, television, radio, telephone, bed, cupboard, electric fan and electric devices).

In our study, the agricultural work are all tasks in relation to the rice cultivation, vegetable planting, animal husbandry, and fish feeding. Latrine can refer to a toilet or a simpler facility used as a toilet, it can be a simple pit, single vault, double vault or more advanced designs, including pour-flush systems or ecological latrines. Use of excreta is the household had used of human and/or animal excreta for fertilising in the field and/or feeding fish. The handling of excreta means that a farmer is emptying or collecting, composting, transporting or applying excreta. The protective measures including, working cloth, face mask, plastic gloves and boots, which farmers used when contact with wastewater or excreta.

An episode of acute diarrhoea was defined as: (i) three or more loose (or watery) stools within 24 hours, regardless of other gastrointestinal symptoms; or (ii) two or more loose stools associated with at least one other symptom of gastrointestinal infection (abdominal pain, cramping, nausea, vomiting, and fever); or (iii) passage of a single loose stool with grossly evident blood and/or mucous [23, 24]. Two independent diarrhoea episodes were separated by at least three days without diarrhoea, and an episode of diarrhoea with duration of 14 days or more was regarded as an episode of persistent diarrhoea [25]. An individual self-reporting sheet was used to monitor the subject’s exposure to wastewater and excreta. The total time of exposure to wastewater and excreta was recorded daily for each study subject; other potential risk factors (e.g., use of protective measures, hand-washing with soap, drinking raw water, and eating raw vegetables) were also recorded.

A prospective case–control study was conducted as part of prospective monitoring of diarrhoeal episodes among all followed subjects in order to assess the relationship between diarrhoea and exposure to excreta and wastewater (i.e., direct contact with human and animal excreta, Nhue River and local pond water). Other potential risk factors of diarrhoeal disease (e.g., personal hygiene aspects, drinking water, food consumption, etc.) were also obtained. The history of exposure was defined as one week prior to the day of diarrhoea occurrence or the day of control interviews. For diarrhoea cases, information was collected on the characteristics of the diarrhoea (i.e., duration of episodes, number of stools per day, characteristics of stool, symptoms, and any related treatment).

Cases were detected and selected by the local health workers by recording weekly morbidity. We used an incidence-density sampling of cases and controls [26], meaning that controls were sampled concurrently among the cohort. Under the incidence-density sampling scheme, a case could end up as a control later on or vice versa, and the control might be selected by chance for more than one case during the follow-up period. When a case was ascertained, a control (the ratio of cases to controls is 1:1) was randomly selected in the population at risk. This control was an individual who did not experience diarrhoea in the previous two weeks, lived in the community, and was from a different household than the case.

A questionnaire was administered to all cases and controls. The questionnaire was developed in English, translated to Vietnamese, back-translated for confirmation and pre-tested in villages close to Hanoi. After adaptation the questionnaire was used in face-to-face interviews by five trained and experienced research assistants to all cases and controls. Principal researchers accompanied each assistant to three individual interviewees for quality control (e.g., utilization of same procedures were used and for quality as being precisely followed). Each interview lasted approximately 45 minutes.

### Data management and analysis

The diarrhoeal disease incidence was calculated for the cohort study over one year of the follow-up period. The days under surveillance for each participant were recorded, allowing the calculation of an exact number of days at risk between episodes of diarrhoeal disease (person-time at risk). The negative binomial regression model was employed to estimate the relative rate (RR) from the incidence data. Generalized Estimating Equations (GEE) were used to account for correlation within individuals and household [27]. For the risk factor analysis, conditional logistic regression was used in univariable and multivariable analyses from the nested case–control study. First, a univariable conditional logistic regression analysis adjusted for age group (16–35 years, 36–55 year, older than 55 years) and sex was carried out to associate potential risk factors with disease outcome (i.e., diarrhoeal episode). Matched odds ratio (OR) and its 95% confidence interval (CI) and P-value were calculated and reported. Then, variables with P < 0.2 in the univariable analysis were included in the multivariable conditional logistic regression analysis. Multivariable analyses were performed to evaluate the effect of the explanatory variables, controlling for the effect of other risk factors [28]. The attributable fraction (AF) in the population was calculated with the assumption that the exposed proportion in the control group (Pe) is that of the whole population. AF was calculated for the OR of each significant variable in the multivariable model using Levin’s formula (equation 1) [29] for assessing the importance of exposure to the population.
1

SES and sanitary conditions in the household were calculated according to an asset-based method [30–32]. In brief, indicator data were defined by principal component analysis (PCA), with missing values being replaced with the mean value of the respective asset; all assets had a dichotomous character. SES and sanitary conditions in the household were categorized into three levels as good, average, and poor according to their cumulative standardized asset scores.

Data were entered into a Microsoft Access data-base, and analysed using STATA 10.1 Software (STATA-Corporation, College Station, TX, USA).

### Ethical considerations

The Ethical Research Committee at the National Institute of Hygiene and Epidemiology (NIHE, number 149/QĐ-VSDTTƯ-QLKH), Vietnamese Ministry of Health and the Ethic Commission of the State of Basel (EKBB, number 139/09) approved the study. Before field work began, the authorities in the Provincial Health Office and the District Health Office were informed on study objectives and procedures and working authorization obtained. Written informed consent was obtained from each individual prior to enrolment.

## Results

### Incidence of diarrhoeal disease

**Figure 2:**
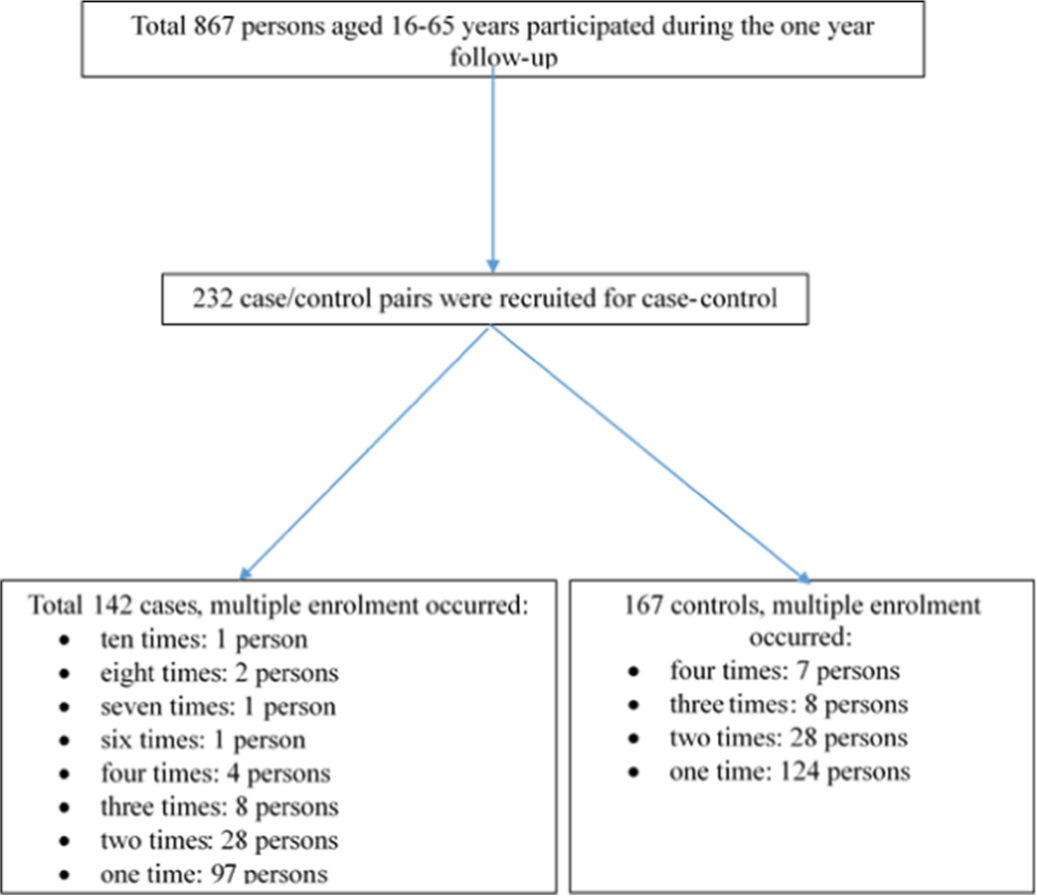
**Case–control flow chart in the study.**

A total of 1,070 persons aged 16–65 years (median age 37 years, 53% females) from 424 households were enrolled in the baseline survey. Among them 867 persons participated during the one year follow-up. Median time of follow-up was 26 weeks (inter-quartile range [IQR] 14–40) resulting in a total 299,222 days. Diarrhoeal diseases were reported by 142 subjects (16%), with a total of 232 episodes (Figure 2). This yields an incidence of 0.28 episodes per person-year at risk (95% CI 0.25 -0.32).

**Figure 3:**
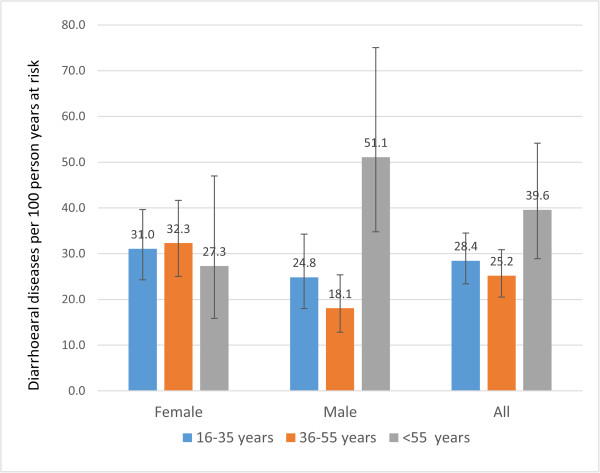
**Diarrhoea incidence by age and gender in 867 adult persons followed for 299,222 person-days, Hanam province, Vietnam, 2009–2010.**

**Figure 4:**
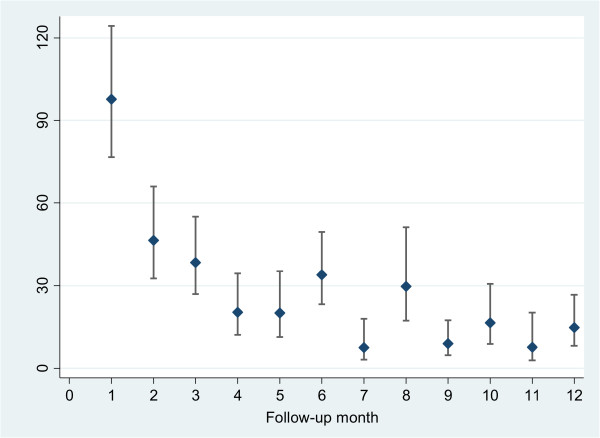
**Monthly diarrhoea incidence in 867 adult persons followed for 299,222 person-days, Hanam province, Vietnam, 2009–2010.**

Figure 3 shows age- and sex-specific incidence. The lowest diarrhoeal incidence was in participants aged 36–55 years (0.25 episodes per person-year at risk, 95% CI 0.21-0.31); followed by aged 16–35 years (0.28 episodes per person-year at risk, 95% CI 0.23-0.35); and aged 56–65 years (0.40 episodes per person-year at risk, 95% CI 0.29-0.54). There was no statistically significant difference in diarrhoeal incidence rates between males and females (RR = 0.83, 95% CI 0.54-1.26). The monthly incidence of diarrhoea is given in Figure 4. The highest diarrhoeal incidence was observed in the first month of our follow-up.

### Characteristics of diarrhoeal diseases

A total of 232 case/control pairs were recruited for the case–control study. One hundred and forty two subjects were cases. Forty-five cases experienced more than one diarrhoeal episode: one person was enrolled as a case ten times; two persons eight times; one person seven times; one person six times; four persons four times; eight persons thrice and 28 persons twice. Of the 167 subjects recruited as controls, seven persons were picked four times, eight persons thrice and 28 persons twice (Figure 2).

The mean duration of a diarrhoeal episode was 2 days (interquartile range: 1–6 days). Of all episodes, 3 (1%) had duration of ≥ 7 days, and none of the 232 episodes was persistent. The mean number of stools per day was 3.3 (interquartile range: 2–5 stools). Eight stools (4%) from cases had grossly evident blood, 83 stools (36%) contained mucous and 181 stools (78%) were watery. Study cases reported the abdominal pain (197 cases, 85%); thirst (82, 79%); fatigue (107, 46%); nausea (58, 25%) and fever (26, 11%). In terms of care, self-treatment was most common (104, 45%), followed by consulting pharmacists (74, 32%), private doctor (29, 13%), local health centre (23, 10%) and hospital (2, 1%).

### Risk factors of diarrhoeal episodes

The results of univariable and multivariable conditional logistic regression analysis are presented in Tables 2 and 3, respectively. Among the indicators describing household sanitary and hygiene conditions were the water sources used for drinking and having a member with diarrhoea in a family were associated with diarrhoeal disease. Participants who lived in households using rainwater to drink had a higher risk of diarrhoea than those living in households with tap water in univariable (OR = 3.9, 95% CI 2.0-7.4) and in multivariable analysis (OR = 5.4, 95% CI 2.4-12.1). However, the use of tube well water was not a greater odds of diarrhoea than the use of tap water in both uni- and multivariable analyses (OR = 2.8, 95% CI 0.7-10.9 and OR = 2.2, 95% CI 0.4-12.4, respectively). Therefore, it was not possible to find statistically significant differences. However, there was some indication that the use of tube water was associated with higher risk. Contact with persons with diarrhoea also increased the risk of diarrhoea in univariable (OR = 4.7, 95% CI 2.0-11.3) and in multivariable analysis (OR = 3.7, 95% CI 1.4-10.3). The use of dry latrines (single or double vault) and water-flushed latrines (septic tank or biogas) was not statistically significantly associated with the risks of diarrhoea in comparison with the households without a latrine (OR = 1.4, 95% CI 0.5-3.7 and OR = 1.2, 95% CI 0.4-3.4, respectively). Close contact with domestic animals in household was not associated increasing the risk of diarrhoeal diseases (OR = 1.0, 95% CI 0.7-1.5).

**Table 2:** **Univariable risk factors analysis for adult diarrhoeal disease in 232 cases and 232 controls in Hanam province, Vietnam, 2009-2010**

Risk factors		Case	Control	OR *	95% CI	P-value
		N (%)	N (%)			
**1. Demographic characteristics**						
Sex (adjusted for age)						
	Female	135 (58)	134 (58)	Reference		
	Male	97 (42)	98 (42)	0.9	0.6-1.4	0.74
Age groups (in years, adjusted for sex)						
	16-35	100 (43)	101 (44)	Reference		
	36-55	93 (40)	101 (44)	0.9	0.6-1.4	0.73
	56-65	39 (17)	30 (13)	1.3	0.8-2.4	0.31
Educational level						
	Pre-school & primary school	79 (34)	74 (32)	Reference		
	Secondary & tertiary education	153 (66)	158 (68)	0.9	0.6-1.4	0.71
Occupation						
	Non-agricultural work	45 (19)	58 (25)	Reference		
	Agricultural work	187 (81)	174 (75)	1.4	0.9-2.3	0.13
Household’s socio-economic status						
	Poor	68 (29)	68 (29)	Reference		
	Average	85 (37)	70 (30)	1.3	0.1-2.2	0.26
	Good	79 (34)	94 (41)	0.9	0.6-1.5	0.76
**2. Household sanitary and hygiene conditions**						
Type of latrine in the household						
	No latrine	7 (3)	9 (4)	Reference		
	Dry latrine	144 (62)	134 (58)	1.4	0.5-3.7	0.57
	Water-flushed latrine	81 (35)	89 (38)	1.2	0.4-3.4	0.74
Source to drink water						
	Tap water	15 (7)	45 (19)	Reference		
	Rainwater	212 (91)	181 (78)	3.9	2.0-7.4	<0.01
	Tube well water	5 (2)	6 (3)	2.8	0.7-10.9	0.14
Close contact with animals in household						
	No	113 (49)	114 (49)	Reference		
	Yes	119 (51)	118 (51)	1.0	0.7-1.5	0.88
Contact with person with diarrhoea						
	No	204 (88)	225 (97)	Reference		
	Yes	28 (12)	7 (3)	4.7	2.0-11.3	<0.01
**3. Exposed to human and animal excreta**						
Composting of human excreta in the household						
	Compost > 3 months	44 (19)	66 (29)	Reference		
	≤ 3 months	188 (81)	166 (72)	1.8	1.2-2.8	0.01
Use of human excreta for application in field						
	No	131 (57)	137 (59)	Reference		
	Yes	101 (43)	95 (41)	1.1	0.7-1.6	0.69
Handling human excreta in field work						
	No	214 (92)	228 (98)	Reference		
	Yes	18 (8)	4 (2)	5.1	1.7-15.3	<0.01
Use of animal excreta as fertiliser in the fields						
	No	122 (53)	157 (68)	Reference		
	Yes	110 (47)	75 (32)	1.9	1.3-2.7	<0.01
Handling animal excreta in field work						
	No	145 (63)	175 (75)	Reference		
	Yes	87 (38)	57 (25)	2.0	1.3-3.0	<0.01
**4. Exposure to Nhue River water and pond water**						
Use Nhue River water to irrigate fields						
	No	13 (6)	23 (10)	Reference		
	Yes	219 (94)	209 (90)	1.9	0.9-3.8	0.08
Direct contact with Nhue River water during field work						
	No	149 (64)	171 (74)	Reference		
	Yes	83 (36)	61 (26)	1.7	1.1-2.6	0.02
Close contact with local pond water (washing, fishing)						
	No	173 (75)	202 (87)	Reference		
	Yes	59 (25)	30 (12)	2.4	1.5-4.0	<0.01
**5. Habits of personal hygiene**						
Use of protective measures (gloves, boots and face mask) at work						
	Yes	67 (29)	90 (39)	Reference		
	No	165 (71)	142 (61)	1.6	1.1-2.4	0.02
Hand washing with soap in general						
	Frequently	35 (15)	71 (30)	Reference		
	Sometime	62 (27)	57 (25)	2.2	1.3-3.8	<0.01
	Never or rarely	135 (58)	104 (45)	2.7	1.6-4.3	<0.01
Eating raw vegetables the day before						
	No	185 (80)	208 (90)	Reference		
	Yes	47 (20)	24 (10)	2.6	1.5-4.6	<0.01
Eating leftover foods from day before						
	No	86 (37)	127 (55)	Reference		
	Yes	146 (63)	105 (45)	2.1	1.5-3.1	<0.01
Drinking raw water the day before						
	No	194 (84)	202 (87)	Reference		
	Yes	38 (16)	30 (13)	1.4	0.8-2.3	0.25

OR *: matched odds ratio, derived from univariable conditional logistic regression analysis adjusted for age and sex; CI: confident interval.

**Table 3:** **Multivariable risk factors analysis for adult diarrhoeal disease in 232 cases and 232 controls in Hanam province, Vietnam, 2009-2010**

Determinants		OR *	95% CI	AF**	% exposure among controls
Agricultural activities					
	Yes *versus* No	1.1	0.6-2.0	0.04	75
Source of drink water (*versus* tap water)					
	Rainwater	5.4	2.4-12.1	0.77	78
	Tube well water	2.2	0.4-12.4	0.03	3
Contact with person with diarrhoea					
	Yes *versus* No	3.7	1.4-10.3	0.08	3
Composting of human excreta in the household					
	≤ 3 months *versus* > 3 months	2.4	1.4-4.3	0.51	72
Handling human excreta in field work					
	Yes *versus* No	5.4	1.4-21.1	0.07	2
Use of animal excreta as fertiliser in the fields					
	Yes *versus* No	1.6	1.0-2.6	0.16	32
Handling animal excreta in field work					
	Yes *versus* No	3.3	1.8-6.0	0.36	25
Use of Nhue River water to irrigate fields					
	Yes *versus* No	1.0	0.4-2.5	0.00	90
Direct contact with Nhue River water during field work					
	Yes *versus* No	2.4	1.2-4.7	0.27	26
Close contact with local pond water					
	Yes *versus* No	2.3	1.2-4.3	0.14	13
No use of protective measures at work					
	Yes *versus* No	6.9	3.5-13.9	0.78	61
Eating raw vegetables the day before					
	Yes *versus* No	2.4	1.2-4.6	0.12	10
Eating leftover foods from day before					
	Yes *versus* No	1.1	0.7-1.8	0.06	45
Handwashing with soap in general (*versus* frequently)					
	Sometime	2.5	1.3-4.9	0.27	25
	Never or rarely	3.3	1.8-6.3	0.51	45

OR*: matched odds ratio derived from multivariable conditional logistic regression analysis and adjusted for age groups and sex.

CI: confidence interval.

AF **: attributable fraction in the population.

Composting of human excreta for less than 3 months was associated with the risk of diarrhoeal disease in both uni- and multivariable analysis (OR = 1.8, 95% CI 1.2-2.8 and OR = 2.5, 95% CI 1.4-4.3, respectively). Household use of human excreta for application in the field was not associated with diarrhoeal disease (OR = 1.1, 95% CI 0.7-1.6). However, higher risk for diarrhoeal episodes was observed in people who had been handling human excreta in field work than those who had not in univariable (OR = 5.1, 95% CI 1.7-15.3) and in multivariable analysis (OR = 5.4, 95% CI 1.4-21.1). The risk of diarrhoeal diseases was statistically significantly associated with the use of animal excreta as fertiliser for application in field in both uni- and multivariable analysis (OR = 1.9, 95% CI 1.3-2.7 and OR = 1.6, 95% CI 1.0-2.6, respectively). In both uni- and multivariable analyses, people who had been handling animal excreta in field work had greater risk of diarrhoeal diseases than those who had not (OR = 2.0, 95% CI 1.3-3.0 and OR = 3.3, 95% CI 1.8-6.0, respectively).

Direct contact with Nhue River water during field work resulted in a risk increase for diarrhoea the uni- (OR = 1.7, 95% CI 1.1-2.6) and multivariable analysis (OR = 2.4, 95% CI 1.2-4.7). Close contact with local pond water (i.e., washing clothes, fishing) was statistically significantly associated with an increased the risk of diarrhoea, also in both analyses (univariable: OR = 2.4, 95% CI 1.5-4.0; multivariable OR = 2.3, 95% CI 1.2-4.3). The use of Nhue River water to irrigate fields was not a risk factor increased the risk of diarrhoea in univariable (OR = 1.9, CI 0.9-3.8) and in multivariable analysis (OR = 1.0, 95% CI 0.4-2.5).

The present study was showed that the personal hygiene practices were change the risk of diarrhoea. No use of personal protective measures during field work (i.e. gloves and boots) increased the risk for diarrhoea in univariable (OR = 1.6, 95% CI 1.1-2.4) and in multivariable analysis (OR = 6.9, 95% CI 3.5-13.9). Omitting hand washing was significantly associated with risk of diarrhoea: people who rarely and sometimes washed their hands with soap had a large increase of odds diarrhoea (OR = 3.3, 95% CI 1.8-6.3 and OR = 2.5, 95% CI 1.3-4.9, respectively) compared to those who frequently washed their hands with soap. Eating raw vegetables the day before was statistically significantly associated with an increased risk of diarrhoea (OR = 2.4, 95% CI 1.2-4.6). Diarrhoea was also associated with the consumption of raw water and leftover foods from the day before (OR = 1.4, 95% CI 0.7-2.9 and OR = 1.1, 95% CI 0.7-1.8, respectively) but was not statistically significant.

In none of the analysis was household’s SES associated with diarrhoeal disease (Tables 2 and 3). Cases and controls did not differ in educational levels. Furthermore, no statistically significantly difference was found in occupation (OR = 1.4, 95% CI 0.9-2.3). Approximately three quarters of both groups were farmers (81% of cases *versus* 75% of controls, P = 0.15). Diarrhoeal disease was not associated with educational level (OR = 0.9, 95% CI 0.6-1.4).

Analysis of the AF in the population (Table 3) showed that the lack of protective measures at work was the principal risk factor. It explain about 78% of diarrhoeal episodes in our population, followed by the use of rainwater for drinking (77%); composting human excreta less than 3 months (51%); never or rarely washing hands with soap (51%); handling animal excreta in field work (36%); direct contact with Nhue River water during field work (27%); close contact with local pond water (14%); eating raw vegetables (12%); close contact with person having a diarrhoea (8%); and handling human excreta in field work (7%).

## Discussion

In the rural agricultural communities in Northern Vietnam, we assessed the incidence of diarrhoeal disease and its risks using a nested case–control approach. We found that the diarrhoeal incidence in adult farmer was lower than the global estimate for developing regions [33]. The handling practices of wastewater, human and animal excreta in agriculture, as well as poor personal hygiene practices such as the lack of protective measures, infrequent hand washing with soap, and consumption of unsafe water or raw vegetables were associated with a high risk of diarrhoeal episodes.

Participants who were in direct contact with water from Nhue River and local ponds during field work had 2.4- and 2.3-fold greater risk of diarrhoeal disease respectively than those who were not. Our results are similar to those from the other studies in Hanoi and in Mexico, where the farmers and their families exposed to wastewater had an excess risk of diarrhoeal disease [16, 17, 34]. In our study, 27% of diarrhoeal episodes could be explained by exposure to Nhue River water; similar results were found by Blumenthal and colleagues [16] who observed that diarrhoeal disease was attributable to raw-wastewater exposure in the dry season. An earlier study in Hanoi indicated that wastewater exposure accounted for 35% of diarrhoeal episodes [17]. We explained the considerable AF of exposure to wastewater the farmers’ frequent exposure to wastewater during different agricultural activities (e.g. soil preparation, planting, fertilising, irrigating, excreta application, harvesting, fish feeding and catching). In addition, we observed during the field visit, farmers did not wear protective measures while doing field work. The reasons could be explained that gloves and boots were not practical to wear and it is very difficult to walk on the narrow paths along the rice fields, and also wear protective measures were inconvenience for farmers during work. In principle, farmers knew they should use protective measures; but in practice, they did not apply this knowledge [35].

In the present study, the risk of diarrhoeal disease was substantially associated with the handling practices of excreta in agriculture. Human excreta composted less than 3 months before fertilising was associated with a risk of diarrhoeal diseases (OR = 2.4, 95% CI 1.4-4.3) and 51% of the diarrhoeal episodes could be explained by that factor. It seems that the composting procedure does not fully comply with the composting guidelines set by the Vietnamese Ministry of Health which imposes a minimum of 6 months [36]. Many intervention studies demonstrated that improving the disposal of human excreta has been effective in reducing risks of diarrhoeal disease up to 36% [37–39]. Our finding further underlines that the safe composting of excreta should be intensively promoted in our setting. As indicated by Jensen and colleagues [40] approximate composting for a duration 4 months under the conditions of high pH and temperature and low moisture could provide a safe compost product to be used for agricultural application. This composting duration was shown to destroy enteric pathogens, thereby reducing the risk for diarrhoea.

People handling human and animal excreta in field work had 5.4- and 3.3-fold higher risk for a diarrhoeal episode respectively than those who did not have contact. However, handling human excreta in agricultural work accounted for only 7% of diarrhoeal cases, whereas handling animal excreta explained 36% of the cases. This corresponds with a larger number of farmers handling animal excreta in field work (25%) in comparison with human excreta (2%) in the communities. The occurrence of diarrhoeal disease was not associated with type of latrine used in the household. A similar observation was made in Ethiopia [41], the mere latrine utilization did not impact the occurrence of childhood diarrhoeal disease.

Our study shows that the risk of diarrhoeal disease was significantly associated with the use of rainwater for drinking in the household, accounting for 77% of diarrhoeal episodes. Our results contradict with previous studies in Kenya and Vietnam, which reported that use of rainwater reduced diarrhoeal risks [42, 43], and also consumption of rainwater did not increase the risk of gastroenteritis among children in South Australia [44]. The most probable explanation for our observation is that it is related to harvest and storage of rainwater. Instead, we observed during household’s visits that roofs and gutters collected rainwater with a sludge layer, which may contribute to favourable growth conditions for microorganisms. It may be a consequence of domestic animals (e.g. chickens and birds) that travel and defecate on the roof and those potential pathogens may be growing in the rainwater collection system. Poor hygiene in storing rainwater in and abstracting rainwater from tanks or at the point of use can also represent a health concern [45]. Otherwise, higher sediments in tanks can provide nutrients for microbes to survive and proliferate [46]. Presence of faecal indicator bacteria in rainwater suggests contamination with faeces, signifying that pathogens, such as *Campylobacter, Salmonella, Vibrio, Cryptosporidium, Giardia,* and enteric viruses, may also be present in the rainwater [45]. As documented by Daoud and colleagues [47], stored rainwater was significantly contaminated with bacteria (67% of rainwater samples were contaminated with faecal coliforms) resulting in significant human health risk from infectious diseases. In our study area, the rainwater was stored in above ground containers which either did not have a lid or had one that was not frequently closed; we also observed that family members usually collecting water from the tank by bare hand with an iron or rubber bucket, which was placed on the ground. This practice could be caused occurrence of pathogens in the rainwater tank. In addition, it might be due to stochastic effects, because the observed number of persons using tap water to drink was small in the study area (6%).

The lack of protective measures (i.e. gloves, boots and face mask) while doing field work and the lack of frequent hand washing with soap had a substantial increase in risk of diarrhoeal disease. These factors are common faecal oral routes in the transmission of common enteric pathogens [48]. With a lack of hand washing before eating and after defecation, and especially after contact with a person with diarrhoea, the pathogens can easily be transmitted from person to person [49]. Indeed, personal hygiene practices have been proven to be an important factor in reducing the transmission of infectious diseases and can reduce diarrhoeal diseases by 42-47% [17, 50]. Our study results are in line with another study in Hanoi which showed that having a family member with diarrhoea also increased the risk of diarrhoeal disease [17]. Many types of enteric pathogens (i.e. *Vibrio cholera*, *Shigella* spp., *Salmonella* spp., and rotavirus, present in excreta, and transition through the environment, which can ultimately cause diarrhoea in new hosts [50]. In the agriculture setting, where farmers practice use of excreta for fertilizing in the fields without protective measures, they may be infected pathogens while doing field work.

The diarrhoea odds ratio was higher in people who ate raw vegetables the day before. This is consistent with observations made by Kaindi and colleagues [51], who found that consumption of vegetables poses a greater risk for food-borne gastrointestinal diseases, whereas, the consumption of boiled milk, washing of hands with soap and presence of proper drainage system had protective effects. It may be interpreted that the vegetables grown in fields irrigated with wastewater were contaminated with faeces, as indicated by high concentrations of thermo tolerant coliform and the presence of protozoan parasites [52]. Regarding the consumption of foods, it has been found that the improper storage of food for later consumption is a risk factor for diarrhoeal diseases [17, 25, 53]. However, our results show that diarrhoeal risk did not differ between people who ate leftover foods and those who not. We observed during household’s visit people usually re-cooked of leftover food at 100°C in 3–5 minutes before eating.

We did not find a link between diarrhoeal disease and participants’ level of education, which goes against the common belief that diarrhoea is associated with lower educational levels. In fact, children’s whose mothers cannot read and write were 1.7 times more likely to get diarrhoea than children’s who mothers were literate [54], whereas, a study was conducted in Ethiopia indicated that mothers educational status had no statistically significant association with acute childhood diarrhoea [55]. However, our study population was relatively well educated. Two-third of our study participants attended secondary or high school and were generally very knowledgeable. A similar observation was made in Saudi Arabia, Vietnam and Uganda [17, 56, 57], these results also indicated that the educational levels was not significantly associated with the risk of diarrhoea. Our results were in line with the previous study in Hanoi that showed no association between diarrhoeal diseases and household’s SES [17]. It could be explained that our study was clearly defined to an agricultural community in a population with a smaller variation SES. This finding came in contrast to the other studies, which found that people had diarrhoea came from lower SES group [58, 59]. This study results were similar to that found by Trang and colleagues [17] conducted in Hanoi, where the different age groups and sex were not associated with the risk of diarrhoeal diseases.

In our study, the diarrhoeal incidence in adults was low (0.28 episodes per persons per year), which was similar to that found as an investigation in Hanoi [17]. Our incidence was higher than what was reported for children in Northern Ghana (0.10 episodes per person per year) and urban and suburban Malaysia (0.24 episodes per person per year) [60, 61]. However, it is much lower than the global estimated incidence of diarrhoeal diseases for age above 5 years in developing regions, which ranged between 0.40 - 0.60 episodes per persons per year [62]. Our results may have been affected by the under-reporting of diarrhoeal episodes because of the unwillingness to participate in the study. We also observed that self-medication was very frequent: 45% of diarrhoea patients treated themselves with the help of family members or neighbours. Moreover, for diarrhoeal disease, local people perceived it as a private issue which was not to be shared to others, especially strangers. On the other hand, number of participants were lost to follow-up or suspended their participations. Toward the end of the follow-up a number of participants were tired of weekly reporting and/or refused to report their health status. This might have resulted in an overall lower number of diarrhoeal episodes reported towards the end of the cohort period. In general farmers did not consider enteric diseases as a serious ill-health. Therefore our study may have underestimated the true diarrhoeal incidence.

The monthly incidence of diarrhoeal disease was low in both the dry season and rainy season. In our study sites, the farmers used water from Nhue River throughout the year for two rice crops as well as the planting of vegetables, and fish feeding. Hence the exposure level to water from Nhue River was almost similar in both seasons, thereby increasing the risk of acquiring infections. This is in line to the other studies, which found that the diarrhoeal incidence did not differ much between two seasons, although the diarrhoeal episodes were more frequent in the dry and cool season [17]. In addition, a study in the Mekong Delta of Vietnam also indicated that there were no consistent associations between diarrhoeal rates and the flood/dry season [63]. Furthermore, Blumenthal and colleagues reported that the untreated wastewater in dry season was a greater risk of enteric infection than in the rainy season [16]. In our study, the peak of diarrhoeal incidence was in August (during the rainy season). This finding could be explained by the fact that in the study sites, this is the period during which people usually empty and compost human excreta as fertilisers for the next crop. It is noted that, excreta contains variety of different pathogens, particularly enteric bacteria such as *E. coli*, *Shigella* spp., *Salmonella* and *Vibrio cholerae* [64]. Therefore, people may have been exposed to the excreted-organisms causing diarrhoeal disease.

The limitation considerations are interviews with questionnaires were used to measure exposures to wastewater, human and animal excreta and other potential risk factors. It is known that questionnaire assessments are associated with considerable recall and reporting bias. Therefore, there is a considerable uncertainty associated with these measures. Nevertheless, for household information such as household SES, water source, latrine use and sanitary condition, general human and animal excreta use, it was not convenient to collect the information repetitively during the household visits, which was considered as a nuisance to a number of participants. Therefore, the household information was only collected once during the baseline cross-sectional surveys and applied to all individuals living in the same families for the analysis of risk factors for the disease outcomes with the individual as the analysis unit. The household variables were used under the assumption that they remained unchanged throughout the cohort study period.

## Conclusion

In an agricultural community of Hanam province, northern Vietnam, the incidence of diarrhoeal diseases in adults was associated with the handling of human and animal excreta, contact with water from Nhue River and local ponds during field work, the lack of use of protective measures, as well as consumption of unsafe water sources and raw vegetables. In the rural areas of Vietnam, the appropriate treatment of wastewater remains limited, and human and animal excreta are widely used. Therefore, to reduce the public health risks related to the use of wastewater and excreta, the safe composting of excreta and use of protective measures while doing field work must be promoted. In addition, improved personal hygiene practices as well as safe water and food consumption should also be promoted.
